# Association of transition cow health with pregnancy per artificial insemination and pregnancy loss in Holstein cows submitted to a Double-Ovsynch protocol for first service

**DOI:** 10.3168/jdsc.2023-0397

**Published:** 2023-09-22

**Authors:** R. Frenkel, P.M. Fricke, A.M.L. Madureira, W. Heuwieser, S. Borchardt

**Affiliations:** 1 Clinic of Animal Reproduction, Faculty of Veterinary Medicine, Freie Universitaet Berlin, Koenigsweg 65, 14163 Berlin, Germany; 2 Department of Dairy Science, University of Wisconsin–Madison, Madison, WI 53706; 3 University of Guelph, Ridgetown Campus, Ridgetown, ON, Canada N0P 2C0

## Abstract

This observational study was conducted to evaluate the effect of transition cow health on pregnancy per artificial insemination (P/AI) and pregnancy loss (PL) in cows submitted to a Double-Ovsynch protocol (DO) for first service. Lactating Holstein cows (n = 15,041) from one commercial dairy farm in northern Germany between January 2015 to December 2021 were enrolled into a modified Double-Ovsynch protocol (GnRH, 7 d later PGF_2α_, 3 d later GnRH, 7 d later GnRH, 7 d later PGF_2α_, 24 h later PGF_2α_, 32 h later GnRH, and 16 to 18 h later timed artificial insemination) for first service at 72 ± 3 d in milk. Pregnancy was diagnosed at 32 and 60 d post-AI via transrectal ultrasonography. Pregnancy loss was defined as the proportion of cows diagnosed pregnant 32 d post-artificial insemination that were diagnosed nonpregnant 60 d post-artificial insemination. Health-related events (i.e., milk fever [MF], hyperketonemia [KET], retained fetal membranes [RFM], metritis, mastitis, left displaced abomasum [LDA]) were assessed by farm personnel using standard operating procedures. Multivariable logistic regression was used for testing potential associations between transition cow health event occurrence and outcome variables, including P/AI and PL. Three separate models were built for cows in first lactation, second lactation, and ≥third lactation. Overall, 20.0% (885/4,430), 34.9% (1,391/3,989), and 53.9% (3,570/6,622) of cows had at least one transition cow health event for first, second, and ≥third lactations, respectively. The most prevalent transition cow health event for first-lactation cows was metritis (10.7%; [473/4,430]), whereas second-lactation cows suffered mostly from mastitis (16.6%; [664/3,989] and KET (16.6%; [661/3,989]), and cows with ≥third lactations were mostly affected by KET (33.2%; [2,198/6,622]). We observed a negative association between inflammatory disorders (i.e., RFM, metritis, mastitis) and P/AI in all cows irrespective of parity. Metabolic disorders (i.e., MF, KET, LDA) were negatively associated with P/AI only in multiparous cows. Irrespective of parity, only uterine diseases (i.e., RFM, metritis) were significantly associated with PL. These results show that enrolling cows into a fertility protocol, such as DO, cannot overcome the carryover effects of inflammatory and metabolic disorders on P/AI and PL and highlight the importance of optimizing transition cow health as a prerequisite for achieving high fertility in a DO protocol.

## Highlights

• Inflammatory disorders (i.e., retained fetal membranes, metritis, mastitis) had a negative impact on P/AI, irrespective of parity.
• Metabolic disorders (i.e., milk fever, hyperketonemia, displaced abomasum) were negatively associated with P/AI only in multiparous cows.
• Irrespective of parity, uterine-related disorders (i.e., retained fetal membranes, metritis) were associated with pregnancy loss.
• A fertility protocol, such as Double-Ovsynch, cannot overcome the carryover effects of inflammatory and metabolic disorders on P/AI and pregnancy loss.

## Graphical Abstract

**Figure:**
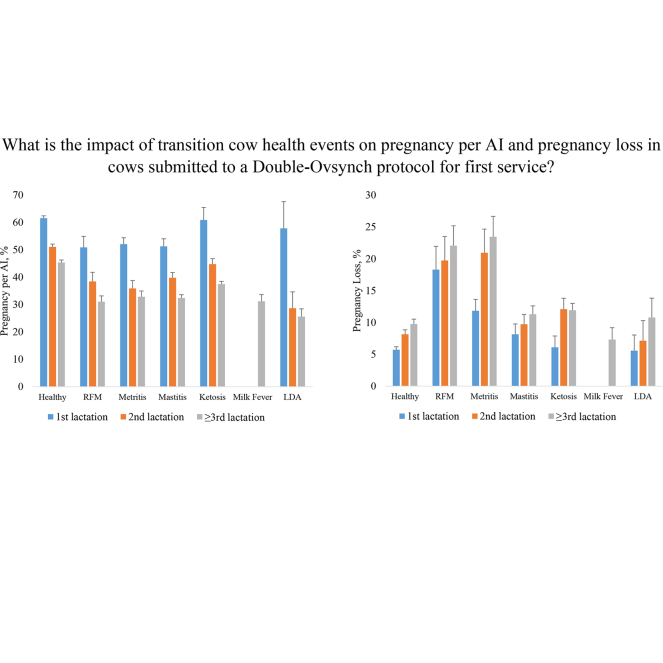
**Summary:** This observational study was conducted to evaluate the effect of transition cow health on pregnancy per artificial insemination (P/AI) and pregnancy loss in cows submitted to a Double-Ovsynch protocol for first service. There was a negative association of inflammatory disorders (i.e., retained fetal membranes, metritis, mastitis) and P/AI in cows submitted to a Double-Ovsynch protocol for first service. Metabolic disorders (i.e., milk fever, ketosis, left displaced abomasum) were negatively associated with P/AI only in multiparous cows. Regarding pregnancy loss, only uterine-related disorders (i.e., retained fetal membranes and metritis) were negatively associated, irrespective of parity. These results highlight the importance of transition cow health as a prerequisite for achieving high fertility in cows submitted to a Double-Ovsynch protocol for first service.

The development of fertility programs over the past decade led to an increase in reproductive performance in the dairy industry (Carvalho et al., 2018; Fricke and Wiltbank 2022). Fertility programs, such as Double-Ovsynch (**DO**) for first timed AI not only increased the AI service rate, but also pregnancy per AI (**P/AI**) compared with cows bred on an AI program based on estrus detection (Fricke and Wiltbank 2022) or a synchronization protocol with prostaglandin (**PGF**; Herlihy et al., 2012). Compared with cows inseminated after detected estrus, cows receiving timed AI after DO have increased P/AI (38.6% vs. 49.0%; Santos et al., 2017). Compared with other presynchronization protocols such as Presynch-Ovsynch, P/AI was greater in cows synchronized with a DO (46.3% vs. 36.8%), with a greater effect in primiparous (52.5% vs. 42.3%) than multiparous cows (40.3% vs. 34.3%; Herlihy et al., 2012). Irrespective of the timed AI protocol used, diseases in early lactation have been associated with reduced P/AI (Santos et al., 2010; Ribeiro et al., 2016) and increased risk for pregnancy loss (**PL**; Santos et al., 2010; Ribeiro et al., 2016). Ribeiro et al. (2016) demonstrated that inflammatory disease before breeding reduced fertilization of oocytes and development to morula, and impaired elongation of early conceptus and secretion of IFN-τ in the uterine lumen. The authors postulated that reduced oocyte competence is a likely reason for carryover effects of diseases on developmental biology, but impaired uterine environment was also shown to be involved. In addition, inflammatory diseases might have indirect consequences on fertility (Ribeiro et al., 2016) such as reduced nutrient intake, increased energy expenditure leading to excessive BW loss, and shifting away nutrients from other physiologic processes, including reproduction (Gifford et al., 2012).

Therefore, the objective of this study was to evaluate risk factors for P/AI and PL in lactating Holstein cows submitted to a DO protocol for first service. We hypothesized that transition cow health events have a detrimental effect on P/AI and PL in cows submitted to a fertility program for first service.

This study was an observational, retrospective cohort study. All experimental procedures were approved by the Institutional Animal Care and Use Committee of the Freie Universität Berlin.

Data from a commercial dairy farm in northern Germany between January 2015 and December 2021 were utilized. The farm had an average (±SD) of 2,872 (±110) calvings per year with an average 305 milk yield of 11,520 kg. Health and breeding records were collected from the on-farm computer system Dairy Comp 305 (Valley Ag Software, Tulare, CA).

Transition cow management was previously described (Venjakob et al., 2019).

Every postpartum cow until 10 DIM was enrolled in this study and was examined daily by the farm personnel following standard operating procedures created by the herd manager. These examinations have been described in detail elsewhere (Venjakob et al., 2021). Between 11 and 30 DIM cows were transferred to the regular milking barn and screened for transition cow health events based on visual observations performed by the farm personnel every morning at 0800 h and on deviations in their daily milk production. The following transition cow health events were considered during the first 30 DIM: milk fever (**MF**), retained fetal membrane (**RFM**), metritis, hyperketonemia (**KET**), and left displaced abomasum (**LDA**). Mastitis was considered during the first 100 DIM. The farm had a long-term history in collaborating with the Clinic for Animal Reproduction, and transition cow health event definitions were based on protocols established with the research team. When cows were recumbent during the first 5 DIM and rose in response to an i.v. Ca infusion, cows were considered as having MF; KET was diagnosed using a cow-side BHB test (Precision Xtra, Abbott Laboratories, Abbott Park, IL); cows were checked for ketosis every Monday and Friday beginning at 2 DIM and a cow was considered hyperketonemic if she had BHB ≥1.2 mmol/L in either of these tests; when percussion of the left flank resulted in tympanic resonance auscultated with a stethoscope, cows were considered to suffer from LDA; when fetal membranes were not expelled by 24 h after calving, cows were diagnosed with RFM; signs of systemic illness (e.g., decreased milk production, dullness, or other signs of toxemia) in combination with an abnormally enlarged uterus, fetid watery red-brown uterine discharge and fever >39.5°C were considered as puerperal metritis (Sheldon et al., 2006); clinical mastitis was defined as visible signs of inflammation in an affected mammary gland (i.e., redness, swelling, pain, or heat) and alterations such as clots, flakes, discoloration, or abnormal consistency of secretions (Vasquez et al., 2017). Udder health was evaluated 2 times daily by farm personnel during regular milking.

Cows were inseminated for first service using only timed AI after submission to a Double-Ovsynch protocol as described by Souza et al. (2008) and modified by Brusveen et al. (2009) as follows: GnRH, 7 d later PGF, 3 d later GnRH, 7 d later GnRH, 7 d later PGF, 24 h later PGF, 32 h later GnRH, and 16 to 18 h later timed AI at 72 ± 3 DIM). At 25 d post-AI, all cows received a GnRH treatment. Seven days later at 32 d post-AI, cows underwent pregnancy diagnosis through transrectal ultrasound (Easi-Scan:GO, IMV Imaging, Bellshill, Scotland). A positive pregnancy diagnosis was considered when a viable embryo and a heartbeat was detected. All pregnancy diagnoses were conducted by trained farm personnel. A second pregnancy diagnosis was performed 60 d post-AI. Pregnancy loss was defined as the proportion of pregnant cows on 32 d post-AI that were found nonpregnant on 60 d post-AI.

Cow ID, parity, calving date, milk yield for the first 100 DIM, and breeding information (i.e., DIM, number of AI, pregnancy outcome) were obtained through the on-farm computer software Dairy Comp 305 (Valley Ag Software, Tulare, CA). All data were transferred to Microsoft Excel (Microsoft Corp., Redmond, WA). All statistical analyses were performed using SPSS for Windows (version 28.0, SPSS Inc., IBM, Ehningen, Germany).

Cow within year was the experimental unit. To account for the fact that observations from cows that were enrolled repeatedly in different years were not independent from each other, parity was used as a repeated measure in the models of parous cows. We evaluated 2 different statistical models to assess the effect of each transition cow health event (i.e., MF, RFM, metritis, LDA, KET, mastitis) on P/AI at 32 d post-AI and PL from 32 to 60 d post-AI separately for primiparous cows (i.e., first lactation), cows in second, and cows in lactation 3 or greater. Model building was conducted as recommended by Dohoo et al. (2009), where each parameter was first analyzed separately in a univariable model. Only parameters resulting in univariable models with *P* ≤ 0.10 were included in the final mixed model. Selection of the model that best fit the data was performed by using a backward stepwise elimination procedure that removed all variables with *P* > 0.10 from the model. The initial model included the following explanatory variables as fixed effects: year of AI; month of AI (1–12), transition cow health (disease vs. no disease). For cows in parity 3, parity (3; 4 or greater) was included in the model as an additional fixed effect.

To account for multiple comparisons, the *P*-value was adjusted using a Bonferroni correction. Variables were declared to be significant when *P* ≤ 0.05. A statistical tendency was declared when *P* was between 0.05 and ≤0.10.

A total of 15,041 Holstein cows (first lactation n = 4,430; second lactation n = 3,989 and ≥third lactations n = 6,622) were included in the final statistical analyses from January 2015 to December 2021.

Disease incidences across parities are summarized in Table 1. Overall, 20.0% (885/4,430), 34.9% (1,391/3,989), and 53.9% (3,570/6,622) of cows had at least one transition cow health event for first, second, and ≥third lactations, respectively. In first-lactation cows the most prevalent transition cow health event was metritis (10.7%; [473/4,430]). Cows in second lactation suffered mostly from mastitis (16.6%; [664/3,989] and KET (16.6%; [661/3,989]). Cows in ≥third lactations were mostly affected by KET (33.2%; [2,198/6,622]).

**Table 1:** Frequency distributions (%) for health-related events by parity for cows (n = 15,041) synchronized with a Double-Ovsynch protocol

Health status	First lactation (n = 4,430)	Second lactation (n = 3,989)	≥Third lactation (n = 6,622)
Healthy	80.0	65.1	46.1
Type of health problem^1^			
Retained fetal membrane	3.6	5.2	7.2
Metritis	10.7	6.5	7.1
Clinical mastitis	7.6	16.6	22.0
Milk fever	0.0	0.2	4.8
Hyperketonemia	2.8	16.6	33.2
Left displaced abomasum	0.6	1.1	2.9

1 Retained fetal membrane = fetal membranes were not expelled by 24 h after calving; metritis = sign of systemic illness (e.g., decreased milk production, dullness, or other signs of toxemia) in combination with an animal with an abnormally enlarged uterus, fetid watery red-brown uterine discharge, and fever >39.5°C; clinical mastitis = visible signs of inflammation in an affected mammary gland (i.e., redness, swelling, pain, or heat) and alterations such as clots, flakes, discoloration, or abnormal consistency of secretions; milk fever = when cows were recumbent during the first 5 DIM and rose in response to an i.v. Ca infusion; hyperketonemia = blood BHB concentrations ≥1.2 mmol/L using a cow-side BHB test; left displaced abomasum = percussion of the left flank resulted in tympanic resonance auscultated with a stethoscope.

The association of transition cow health events and P/AI at 32 d post-AI is summarized in Table 2. For healthy cows (i.e., unaffected by transition cow health events), P/AI decreased (*P* = 0.001) as parity increased and was 61.6%, 51.1%, and 45.5% for cows in first, second, and ≥third lactations, respectively. In addition, P/AI differed by month of AI across all models (*P* < 0.05) with reduced P/AI during summer months (i.e., July and August). The herd level P/AI at d 32 post-AI over the entire period of study was 48.5% (2016: 45.6 ± 1.2%; 2017: 45.4 ± 1.1%; 2018: 48.7 ± 1.1%; 2019: 52.7 ± 1.1%; 2020: 48.5 ± 1.0%; 2021: 47.1 ± 1.1%). Year of AI was only associated with P/AI in multiparous cows (*P* < 0.05). For cows in second lactation P/AI differed across years (2016: 44.0 ± 2.7%; 2017: 45.1 ± 2.6%; 2018: 51.7 ± 2.5%; 2019: 45.6 ± 2.5%; 2020: 46.2 ± 2.4%; 2021: 44.3 ± 2.3%). For cows in ≥third lactation P/AI differed across years (2016: 35.4 ± 1.8%; 2017: 36.0 ± 1.7%; 2018: 38.3 ± 1.6%; 2019: 46.0 ± 1.7%; 2020: 42.5 ± 1.6%; 2021: 42.2 ± 1.6%).

**Table 2:** Association of health events in early lactation and pregnancy per AI 32 d post-first AI for cows (n = 15,041) synchronized with a Double-Ovsynch protocol

Health status	First lactation			Second lactation			≥Third lactation		
	n = 4,430^1^			n = 3,989^1^			n = 6,622^1^		
	Pregnant, %	Adjusted OR^2^ (95% CI)	*P*-value	Pregnant, %	Adjusted OR (95% CI)	*P*-value	Pregnant, %	Adjusted OR (95% CI)	*P*-value
Healthy	61.6	1.00		51.1	1.00		45.5	1.00	
Type of health problem^3^									
Retained fetal membrane	50.9	0.68 (0.493–0.937)	0.019	38.5	0.67 (0.497–0.89)	0.006	31.1	0.65 (0.528–0.79)	0.001
Metritis	52.1	0.69 (0.573–0.844)	0.001	35.9	0.59 (0.449–0.769)	0.001	32.9	0.71 (0.578–0.864)	0.001
Clinical mastitis	51.3	0.68 (0.542–0.853)	0.001	39.8	0.67 (0.565–0.799)	0.001	32.4	0.65 (0.572–0.733)	0.001
Milk fever	—	—	—	72.2	2.83 (0.723–1.028)	0.178	37.5	0.66 (0.754–0.934)	0.001
Hyperketonemia	60.9	1.04 (0.712–1.525)	0.847	44.8	0.86 (0.639–19,539)	0.098	31.2	0.84 (0.516–0.838)	0.001
Left displaced abomasum	57.9	0.92 (0.425–2.044)	0.824	28.7	0.43 (0.212–0.837)	0.012	25.6	0.50 (0.358–0.691)	0.001

1 Number of cows.

2 OR = odds ratio. Estimates were derived from a generalized linear mixed model that was calculated separately for cows in first, second, and ≥third lactations.

3 Retained fetal membrane = fetal membranes were not expelled by 24 h after calving; metritis = sign of systemic illness (e.g., decreased milk production, dullness, or other signs of toxemia) in combination with an animal with an abnormally enlarged uterus, fetid watery red-brown uterine discharge, and fever >39.5°C; clinical mastitis = visible signs of inflammation in an affected mammary gland (i.e., redness, swelling, pain, or heat) and alterations such as clots, flakes, discoloration, or abnormal consistency of secretions; milk fever = when cows were recumbent during the first 5 DIM and rose in response to an i.v. Ca infusion; hyperketonemia = blood BHB concentrations ≥1.2 mmol/L using a cow-side BHB test; left displaced abomasum = percussion of the left flank resulted in tympanic resonance auscultated with a stethoscope.

In first-lactation cows diagnosed with RFM (odds ratio [**OR**] = 0.68), metritis (OR = 0.69), or mastitis (OR = 0.68) P/AI at 32 d post-AI was reduced compared with cows without the particular inflammatory disorder. Hyperketonemia as well as LDA did not affect P/AI at 32 d post-AI in first-lactation cows. Cows in second lactation that had RFM (OR = 0.67), metritis (OR = 0.59), mastitis (OR = 0.67), or LDA (OR = 0.43) had reduced P/AI at 32 d post-AI compared with cows without the particular disease. For cows in ≥third lactations each disease was negatively associated with P/AI (RFM: OR = 0.65; metritis: OR = 0.71; mastitis: OR = 0.65; milk fever: OR = 0.66; KET: OR = 0.84; LDA: OR = 0.5) compared with cows without the particular disease.

The association of transition cow health events and PL at 60 d post-AI is summarized in Table 3. Irrespective of parity, only uterine diseases were significantly (*P* < 0.05) associated with PL, whereas mastitis, milk fever, KET, and LDA were not associated with PL. In first-lactation cows, cows with RFM (OR = 3.62) and metritis (OR = 2.28) had a greater risk for PL. For cows in second lactation (RFM: OR = 2.62 and metritis: 2.89) and cows in ≥third lactation (RFM: OR = 2.59; metritis: OR = 2.86), there was a similar association of uterine disease and the risk for pregnancy loss at d 60 post-AI. Month of AI and year of AI were not associated with PL (*P* > 0.1) across all models except for cows in ≥third lactations. Year of AI was associated with PL with a slight reduction over these years.

**Table 3:** Association of health events in early lactation and pregnancy loss post-first AI for cows (n = 7,294) synchronized with a Double-Ovsynch protocol

Health status	First lactation			Second lactation			≥Third lactation		
	n = 2,663^1^			n = 1,915^1^			n = 2,716^1^		
	Preg. loss,^2^ %	Adjusted OR^3^(95% CI)	*P*-value	Preg. loss, %	Adjusted OR (95% CI)	*P*-value	Preg. loss, %	Adjusted OR (95% CI)	*P*-value
Healthy	5.7	1.00		8.1	1.00		9.8	1.00	
Type of health problem^4^									
Retained fetal membrane	18.3	3.62 (1.94–6.42)	0.001	19.7	2.62 (1.43–4.58)	0.003	22.1	2.59 (1.69–3.91)	0.001
Metritis	11.8	2.28 (1.46–3.45)	0.001	20.9	2.89 (1.64–4.90)	0.001	23.5	2.86 (1.87–4.28)	0.001
Clinical mastitis	8.1	1.38 (0.77–2.34)	0.259	9.7	1.1 (0.69–1.67)	0.684	11.3	1.11 (0.8–1.51)	0.535
Milk fever	—	—	—	17.1	2.08 (0.98–2.19)	0.544	7.3	0.66 (0.95–1.61)	0.252
Hyperketonemia	6.1	0.99 (0.37–2.25)	0.989	12.1	1.48 (0.11–13.68)	0.063	11.9	1.24 (0.29–1.31)	0.110
Left displaced abomasum	5.6	0.9 (0.05–4.60)	0.921	7.1	0.77 (0.04–4.07)	0.798	10.8	1.04 (0.39–2.29)	0.935

1 Number of cows.

2 Pregnancy (Preg.) loss was defined as the proportion of pregnant cows on 32 d post-AI that were found nonpregnant on 60 d post-AI.

3 OR = odds ratio. Estimates were derived from a generalized linear mixed model that was calculated separately for cows in first, second, and ≥third lactations.

4 Retained fetal membrane = fetal membranes were not expelled by 24 h after calving; metritis = sign of systemic illness (e.g., decreased milk production, dullness, or other signs of toxemia) in combination with an animal with an abnormally enlarged uterus, fetid watery red-brown uterine discharge, and fever >39.5°C; clinical mastitis = visible signs of inflammation in an affected mammary gland (i.e., redness, swelling, pain, or heat) and alterations such as clots, flakes, discoloration, or abnormal consistency of secretions; milk fever = when cows were recumbent during the first 5 DIM and rose in response to an i.v. Ca infusion; hyperketonemia = blood BHB concentrations ≥1.2 mmol/L using a cow-side BHB test; left displaced abomasum = percussion of the left flank resulted in tympanic resonance auscultated with a stethoscope.

The objective of this observational study was to evaluate the association of transition cow health events with P/AI and PL in lactating Holstein cows submitted to a DO protocol for first service.

The proportion of cows with at least one disease event was greater in ≥third-lactation cows (53.9%) compared with second-lactation cows (34.9%) and first-lactation cows (20.0%) in agreement with other studies (Pinedo et al., 2020; Lean et al., 2023). The overall incidence of disease was within the range of US herds previously reported (Pinedo et al., 2020). Multiparous cows suffered more frequently from metabolic disorders (i.e., MF and KET) than primiparous cows. This is also in agreement with several other studies (Pinedo et al., 2020; Lean et al., 2023). By contrast, metritis was more prevalent among primiparous cows as has been reported previously (Venjakob et al., 2019).

Even for cows unaffected by disease, P/AI decreased with increasing parity (first lactation 61.6%; second lactation 51.1%; ≥third lactation 45.4%), whereas PL increased with increasing parity (first lactation 5.1%; second lactation 8.1%; ≥third lactation 9.8%). This is supported by Lean et al. (2023) and illustrates the decrease in reproductive performance in older cows.

Uterine-related disorders were negatively associated with P/AI across all parities. This was also reported in several other studies (Ribeiro et al., 2016; Pinedo et al., 2020). An impaired uterine environment, however, could be a result of long-term consequences of disease on cow metabolism and consequently on uterine histotroph composition, which is critical for conceptus development, implantation, and survival (Ribeiro and Carvalho, 2017). Our study reported that cows affected by clinical mastitis had reduced P/AI irrespective of parity. Inflammatory mediators (e.g., IL-1β, IL-6, IL-8, and TNFα), which occur in the course of infectious diseases, are likely responsible for the reduction in oocyte quality (Bromfield et al., 2015). Their detrimental effect on biological processes, such as conception, development of the early conceptus as well the maternal recognition of pregnancy has been discussed elsewhere in more detail (Ribeiro et al., 2016; Carvalho et al., 2019; Pinedo et al., 2020).

Although metabolic disorders (i.e., LDA and KET) were not associated with P/AI in first-lactation cows, the overall incidence of LDA and KET was low in first-lactation cows. In second-lactation cows, LDA was associated with reduced P/AI and there was a tendency toward reduced P/AI for cows affected by KET. For cows in ≥third lactations, there was a negative association with any of the metabolic disorders (i.e., LDA, KET, MF) and P/AI. Cows affected by KET have increased odds for anovulation (Dubuc et al., 2012) or anestrous (Bretzinger et al., 2023) during the voluntary waiting period. This negative effect might be overcome by using fertility protocols such as DO as it has been reported that submission of anovular cows to a DO protocol can overcome the anovular condition (Herlihy et al., 2012). However, even for cows receiving timed AI for first service using a DO protocol, we observed a negative association of KET and P/AI in multiparous cows. In other studies, association of KET and P/AI at first service have been equivocal (Ospina et al., 2010; McArt et al., 2012). Results from these studies are, however, difficult to compare as they differ in their assessment and definition of KET and in the reproductive management.

As previously described, transition cow disease was also associated with PL. In agreement with Ribeiro et al. (2016), our results show that uterine-related disorders (RFM, MET) were associated with increased PL (*P* < 0.05). The detrimental effect of uterine-related infectious diseases has been associated with impaired embryonic development and maternal recognition of pregnancy (Carvalho et al., 2019). Also, inadequate progesterone concentrations during ovulatory follicle development have a negative impact on oocyte quality (Wiltbank et al., 2016). This resulted in impaired embryonic development in the first week so that 20% to 50% of the pregnant cows underwent pregnancy loss during this period. While this might be a concern for anovular cows inseminated after estrous detection or cows presynchronized with a Presynch-Ovsynch protocol, cows in the present study were submitted to a DO protocol to optimize the hormonal milieu during ovulatory follicle development for the first AI. In contrast to another study (Ribeiro et al., 2016), nonuterine diseases (i.e., mastitis, MF, KET, LDA) were not associated with PL.

In conclusion, we observed a negative association of inflammatory disorders (i.e., RFM, metritis, mastitis) and P/AI in cows synchronized with a DO protocol for first service. Metabolic disorders (i.e., MF, KET, LDA) were negatively associated with P/AI only in multiparous cows. Regarding PL, only uterine-related disorders (i.e., RFM and metritis) were negatively associated irrespective of parity. These results highlight the importance of transition cow health as a prerequisite for achieving high fertility in cows submitted to a DO protocol for first service. Enrolling cows into a fertility protocol such as DO cannot overcome the carryover effects of inflammatory and metabolic disorders on P/AI and PL.
